# A proof-of-concept study of a prototype needle that mitigates intraocular pressure rise following intravitreal injection

**DOI:** 10.1186/s40942-024-00590-0

**Published:** 2024-10-10

**Authors:** Alexander Sverstad, Olav Kristianslund, Goran Petrovski, Morten Carstens Moe, Øystein Kalsnes Jørstad

**Affiliations:** 1 Department of Ophthalmology, Institute for Clinical Medicine, Faculty of Medicine, Oslo University Hospital, University of Oslo, Oslo, Norway; 2 Department of Ophthalmology, University Hospital Centre, University of Split School of Medicine, Split, Croatia; 3 UKLO Network, University St. Kliment Ohridski, Bitola, North Macedonia; 4 Department of Ophthalmology, Oslo University Hospital, Postboks 4950, Nydalen 0424 Oslo, Norway

**Keywords:** Intravitreal injection, Intraocular pressure, Vitreous reflux, Needle, Eye model

## Abstract

**Purpose:**

To determine the feasibility of a prototype needle that enhances vitreous reflux (VR) to control intraocular pressure (IOP) in intravitreal injection (IVI).

**Methods:**

We created an eye model to compare IVI using a standard 30-G needle with four different versions of a 30-G prototype needle with one to four surface grooves that enhanced VR. We injected 50, 70, and 100 µl saline through porcine sclera or 460-µm-thick rubber and measured the peak and 3-second pressure before we extracted the needle and measured the 10-second pressure.

**Results:**

50-µl injection through sclera with the standard needle resulted in mean (SD) pressure of 58.6 (3.8) mmHg at peak, 52.8 (4.7) mmHg at 3 s, and 39.6 (18.0) mmHg at 10 s. The prototype needle lowered the pressure; four grooves resulted in mean (SD) pressure of 29.4 (5.6) mmHg at peak, 22.0 (3.7) mmHg at 3 s, and 7.2 (6.6) mmHg at 10 s. 70-µl and 100-µl injections through sclera with the standard needle resulted in mean (SD) pressure of 68.8 (3.6) and 86.0 (6.0) mmHg at peak. Similar to 50-µl injection, the prototype needle lowered the pressure for 70-µl and 100-µl injections. At 10 s, we observed varying leakage at the injection site for sclera but not for rubber.

**Conclusions:**

The study provides proof of concept for a needle design for which surface grooves enhance VR and counteract the effect of IVI on IOP. The safety and efficacy of the prototype needle must be studied further in a clinical trial.

## Background

Intravitreal injection (IVI) of anti-vascular endothelial growth factor (anti-VEGF) biologics results in a transient increase in intraocular pressure (IOP) and is associated with loss of retinal nerve fibre layer (RNFL) thickness, posing the question of whether it can lead to optic neuropathy [1]. The IOP increase is a direct effect of the injection volume [2]. The standard injection volume is 50 µl, but new biologics with higher volumes raise concern about further aggravation of IOP-related side effects, underscoring the importance of controlling IOP in connection with IVI [3].

Intriguingly, an IVI-related phenomenon can in itself lower IOP: vitreous reflux (VR) at the injection site [4–6]. VR is influenced by injection technique and needle choice. Several studies have shown that orthogonal injections result in more VR than oblique, tunnelled, or bevelled injections [7–13]. Most of these studies have also shown that orthogonal injections lead to lower IOP [7–11]. Other studies have shown that 30-G needles result in more VR than 32-G and 34-G needles [14–16]. Two of these studies have also shown that 30-G needles lead to lower IOP [14, 15]. VR increases in presence of posterior vitreous detachment [5]. On the other hand, VR decreases as the total number of IVI increases, but switching injection quadrant can restore VR, suggesting that repeated injections can restrict VR by traumatizing the conjunctiva and sclera [17, 18].

The fact that injection technique and needle choice can modify the amount of VR raises the question as to whether VR can be systematically utilized to mitigate the effect of IVI on IOP. The purpose of this in-vitro study was to determine the feasibility of a prototype needle for IVI that enhances VR to control IOP.

## Methods

**Fig. 1:**
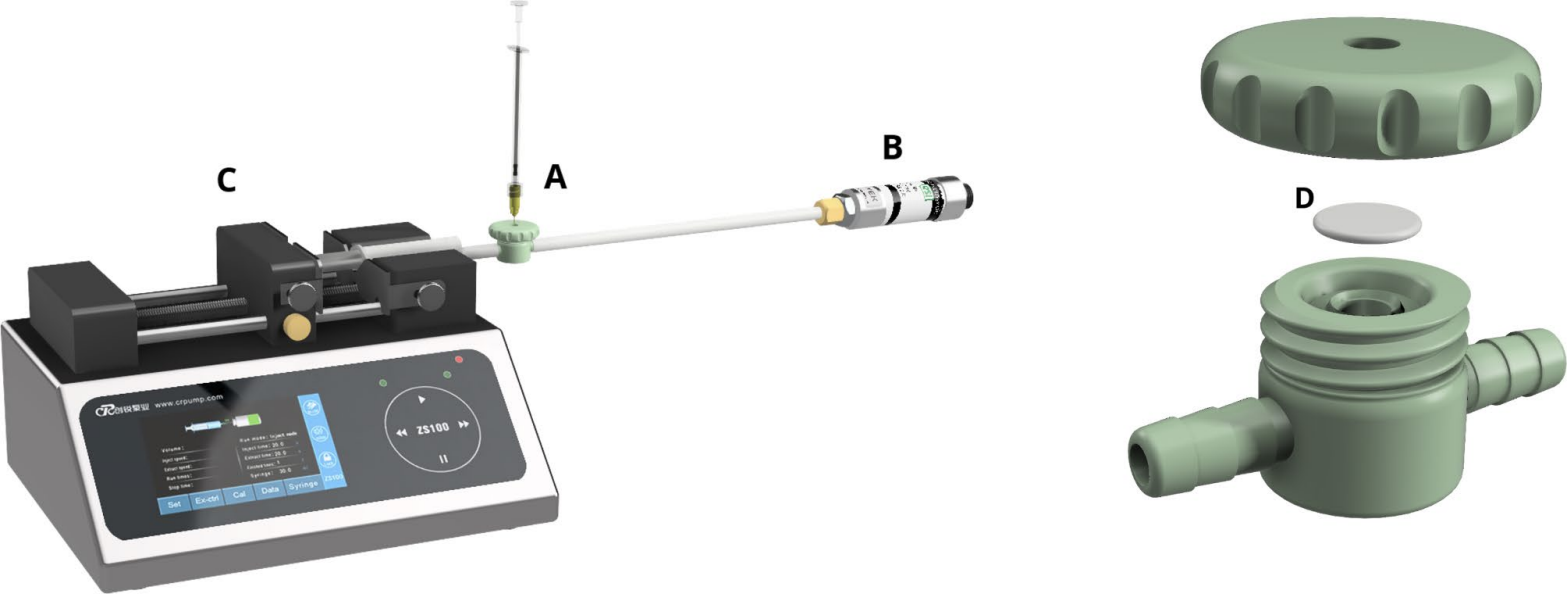
Left: Drawing of the eye model. The model consists of a canister representing the eye (**A**), a dynamic pressure transducer (**B**), and a high-precision syringe pump housing a syringe with synthetic vitreous humour (**C**). Right: Exploded-view drawing of the canister. The central-holed screw cap allows a patch of *pars-plana* porcine sclera (**D**) to be tightly fixated to the canister.

To study the effect of needle design on VR and IOP, we created an eye model that simulated IVI through *pars plana*; the model consisted of a 3D-printed canister (representing the eye) with a central-holed screw cap, which allowed a patch of *pars-plana* porcine sclera to be tightly fixated to the canister (Fig. 1) [19]. We dissected multiple scleral patches from eyes of 6-12-month-old domestic pigs, kept them in glycerol to prevent them from desiccating, and chose the best-preserved patches for the experiments. To remove the glycerol, each scleral patch was washed in saline for five minutes before testing.

A high-precision ZA100 syringe pump (Baoding Chuang Rui Precision Pump Co., Ltd., Baoding, China) housed a syringe containing synthetic vitreous humour with a viscosity of 6–8 cP (Biochemazone, Leduc, Canada). We connected the syringe, the canister, and a GD4200 dynamic pressure transducer (ESI Technology Ltd., Wrexham, UK) and pumped synthetic vitreous humour into the eye model until it reached a pressure of approximately 15 mmHg, similar to physiological IOP. Fluid could then be injected through the scleral patch and into the vitreous-humour-filled canister, creating a pressure increase and, potentially, VR at the injection site. As fluid viscosity depends on temperature and the temperature posterior to the lens is about 32 °C, we performed the experiments at this temperature [20].

**Fig. 2:**
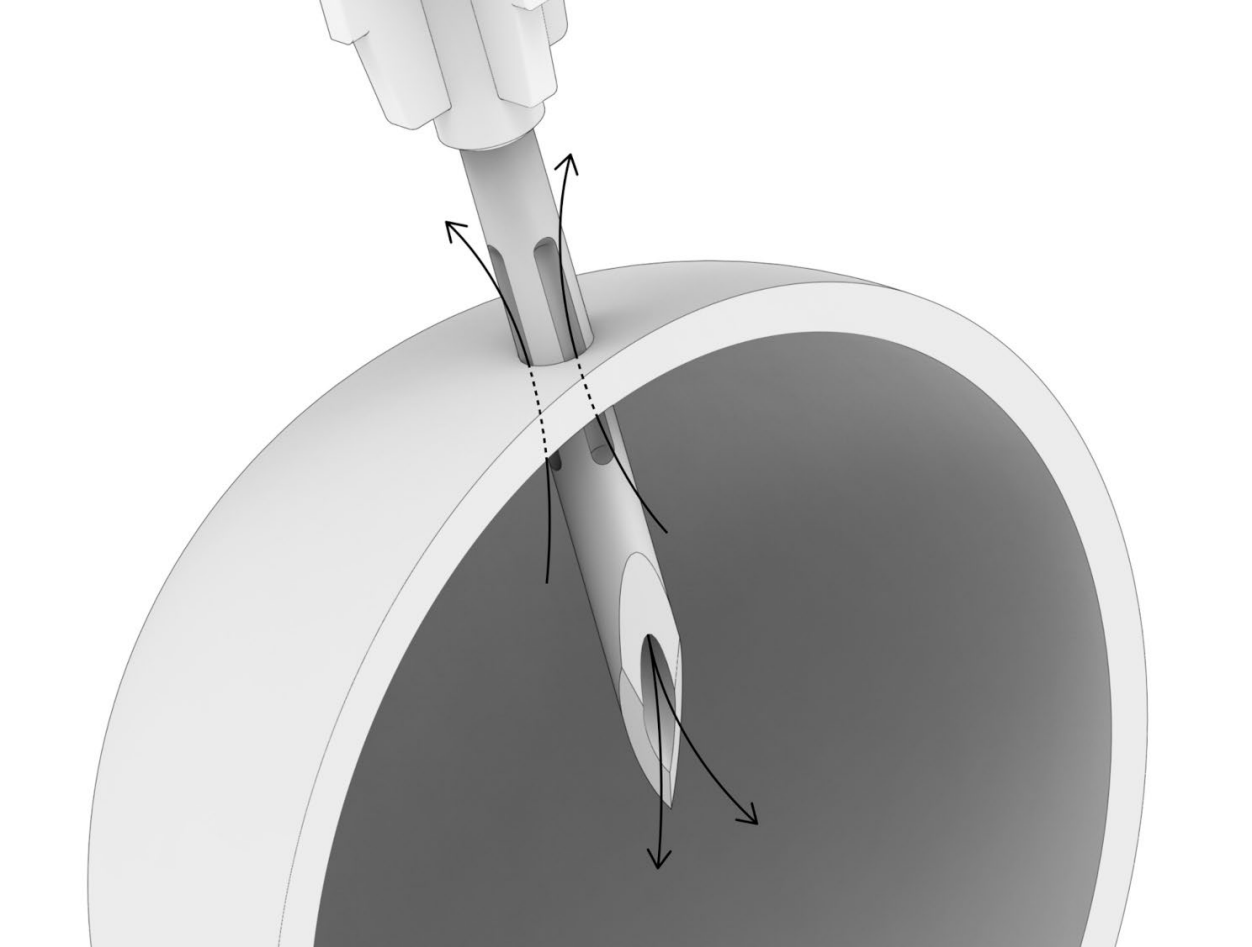
Not-to-scale drawing of the protype needle principle. To enhance vitreous reflux along the needle surface (upwards arrows) simultaneously with injection of fluid through the needle lumen (downwards arrows), the needle has surface grooves along the basal half of its length.

We compared two different needle types in the study: a standard needle and a prototype needle for IVI from SJJ Solutions (The Hague, the Netherlands) (Fig. 2). Both needles had 13-mm length and 30-G diameter. Additionally, the protype needle had surface grooves along the basal half of its length. We tested four versions of the prototype needle with one, two, three, or four grooves. In theory, each groove should enhance VR along the needle surface simultaneously with fluid injection through the needle lumen (Fig. 2). We attached each needle to a 250-µl, high-precision glass syringe (Hamilton, Reno, NV), penetrated the scleral patch orthogonally through the central hole of the cap, and injected the following volumes of isotonic saline into the eye model: (a) 50 µl (standard injection volume), (b) 70 µl (the injection volume of Eylea 8 mg, Bayer, Leverkusen, Germany), and (c) 100 µl (the injection volume of Syfovre, Apellis Pharmaceutical, MA and Izervay, Astellas Pharma, Tokyo, Japan).

We tested the needles in the same order for each injection volume: [1] four-groove prototype [2], three-groove prototype [3], two-groove prototype [4], one-groove prototype, and [5] standard needle. We measured the initial peak pressure and the pressure after 3 s. We then extracted the needle and measured the pressure again after 10 s, before resetting the setup. To account for variability, we repeated the experiment five times for each injection volume and performed each repetition with a new scleral patch. For comparison, we also repeated the experiment with 460-µm-thick rubber patches instead of scleral patches. Because the rubber patches showed less variability in pressure than the scleral patches, we repeated each rubber-patch experiment three times instead of five times.

We used a Deutsches Institut für Normung (DIN) 13,097 test to measure the penetration performance of the prototype needle. In this test a needle is mounted perpendicular to a foil in a testing machine, and the machine then records the penetration characteristics in a load-displacement diagram, which typically displays four different phases: the piercing phase (F0), the cutting phase (F1), the dilatation phase (F2), and the sliding phase (F3) [21].

## Results

**Fig. 3:**
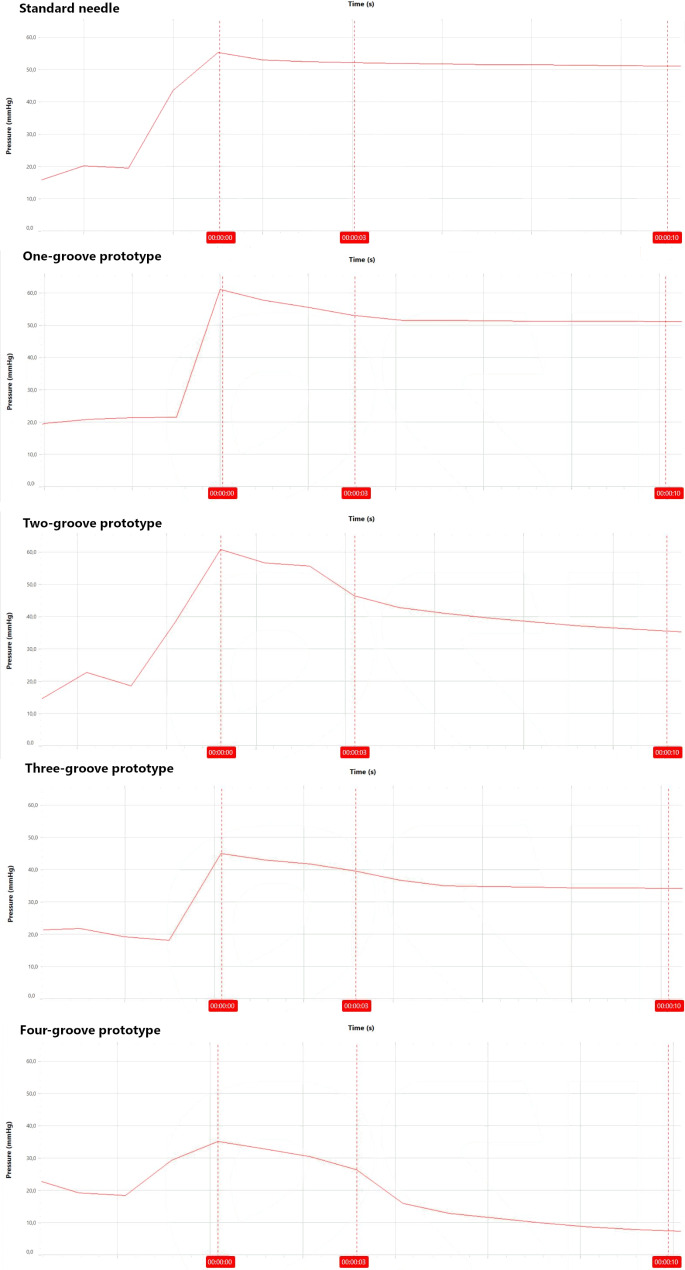
Printouts of the dynamic pressure transducer display during injection of 50 µl isotonic saline through porcine sclera with a standard 30-G needle and prototype needles with one, two, three, or four surface grooves. The needle is extracted after 3 s. Each injection results in an initial pressure peak followed by a pressure drop. Each groove that is added to the prototype needle increases the pressure drop. For the three- and four-groove prototype needles in particular, the peak pressure also decreases.

Figure 3 shows printouts of the dynamic pressure transducer display during injection of 50 µl isotonic saline through porcine sclera with the different needles. Injection with a standard 30-G needle resulted in an initial pressure peak. The pressure then dropped until we extracted the needle after 3 s and continued to fall gradually until the final measurement after 10 s. Injection with the one-groove prototype needle modified this pressure pattern; there was still an initial rise in pressure, but the consecutive pressure drop was more pronounced. Each groove that was added to the prototype needle further increased the pressure drop. For the three- and four-groove prototype needles in particular, the peak pressure also decreased.

**Fig. 4:**
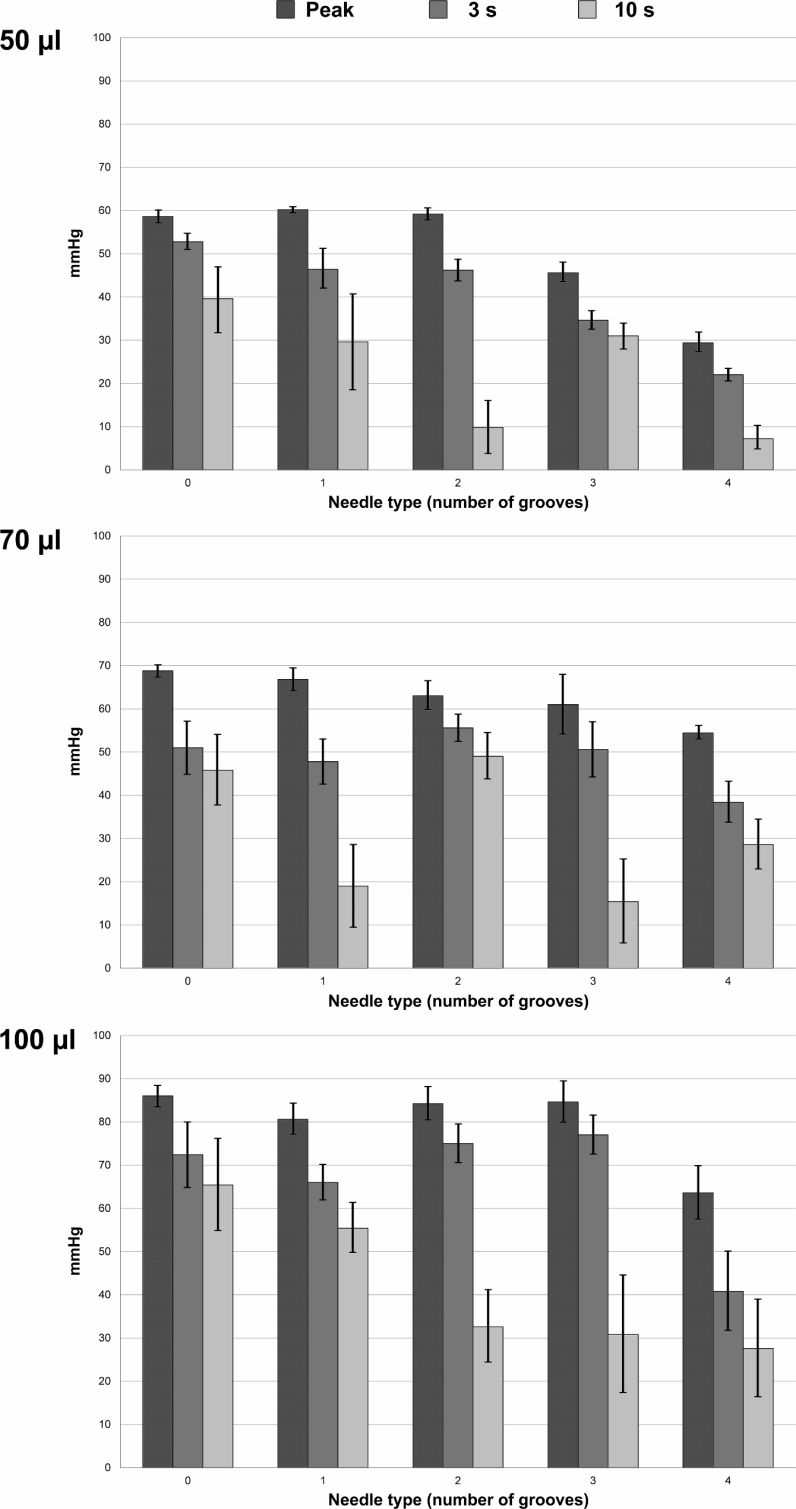
Pressure results (mean pressure and standard deviation) for injection through porcine sclera with different injection volumes and needles.

Injection of 70 µl and 100 µl isotonic saline with a standard 30-G needle increased the initial pressure peak but also the consecutive pressure drop compared to 50-µl injection volume. In a similar way to 50-µl injection volume, the prototype needle modified the pressure for 70-µl and 100-µl injection volumes by increasing the pressure drop. Each groove that was added to the prototype needle further amplified this pattern, and the peak pressure also decreased for the three- and four-groove prototype needles in particular. Table 1 and Fig. 4 show the pressure results for injection through porcine sclera.

**Table 1:** Pressure results for injection through porcine sclera.

**50-µl injection volume**			
Needle type	Peak pressure, mean (SD) mmHg	Pressure after 3 s, mean (SD) mmHg	Pressure after 10 s, mean (SD) mmHg
Standard 30-G	58.6 (3.8)	52.8 (4.7)	39.6 (18.0)
One-groove prototype	60.2 (1.9)	46.4 (10.8)	29.6 (25.9)
Two-groove prototype	59.2 (3.6)	46.2 (6.2)	9.8 (14.6)
Three-groove prototype	45.6 (5.5)	34.6 (5.4)	31.0 (7.3)
Four-groove prototype	29.4 (5.6)	22.0 (3.7)	7.2 (6.6)
**70-µl injection volume**			
Needle type	Peak pressure, mean (SD) mmHg	Pressure after 3 s, mean (SD) mmHg	Pressure after 10 s, mean (SD) mmHg
Standard 30-G	68.8 (3.6)	51.0 (14.6)	45.8 (19.3)
One-groove prototype	66.8 (6.3)	47.8 (12.5)	19.0 (22.5)
Two-groove prototype	63.0 (8.0)	55.6 (7.5)	49.0 (12.7)
Three-groove prototype	61.0 (16.4)	50.6 (15.1)	15.4 (22.9)
Four-groove prototype	54.4 (3.8)	38.4 (11.3)	28.6 (13.7)
**100-µl injection volume**			
Needle type	Peak pressure, mean (SD) mmHg	Pressure after 3 s, mean (SD) mmHg	Pressure after 10 s, mean (SD) mmHg
Standard 30-G	86.0 (6.0)	72.4 (17.9)	65.4 (25.1)
One-groove prototype	80.6 (8.7)	66.0 (9.8)	55.4 (13.8)
Two-groove prototype	84.2 (9.2)	75.0 (10.7)	32.6 (19.8)
Three-groove prototype	84.6 (11.4)	77.0 (10.8)	30.8 (31.9)
Four-groove prototype	63.6 (14.7)	40.8 (21.6)	27.6 (26.5)

**Fig. 5:**
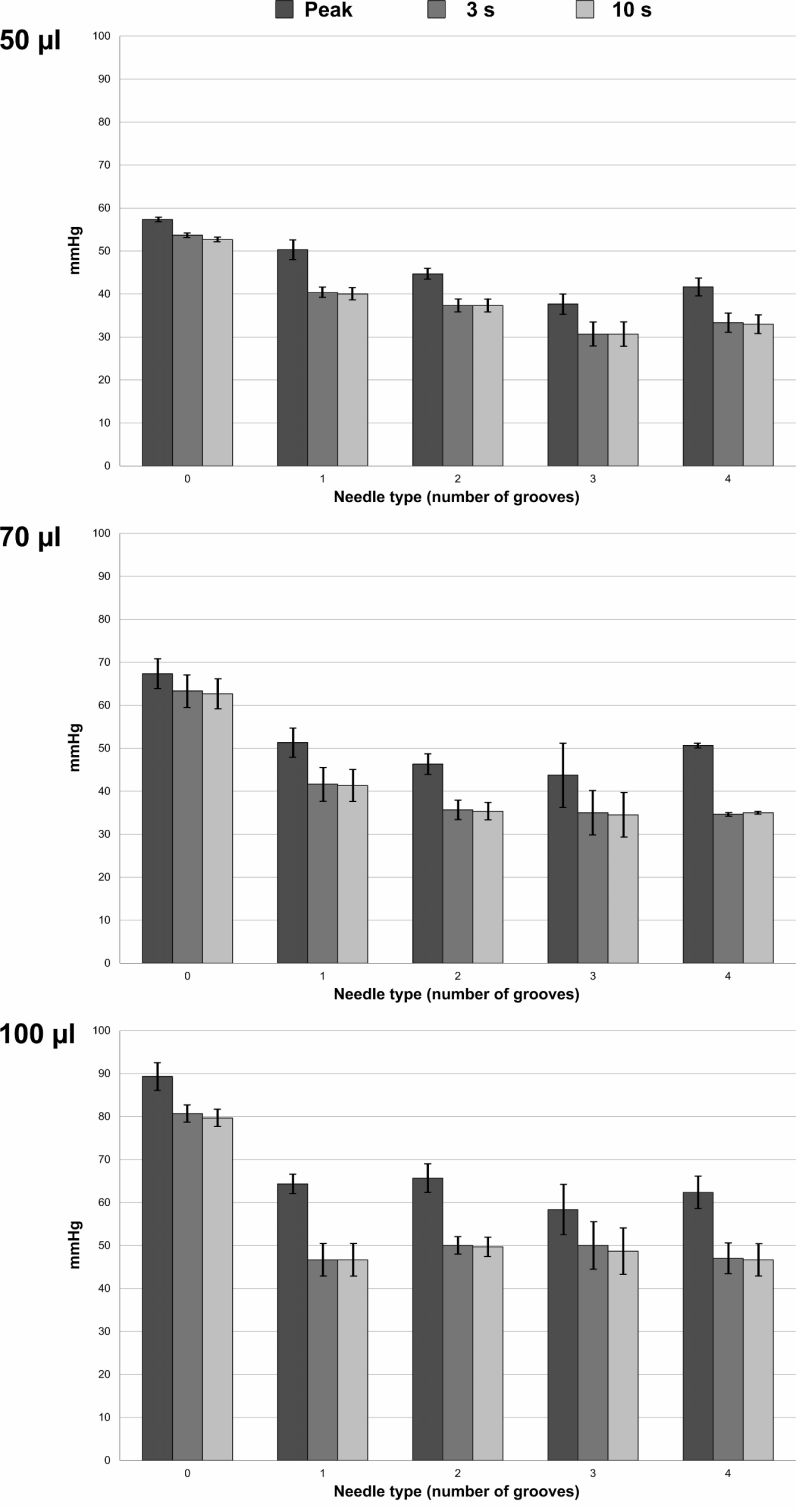
Pressure results (mean pressure and standard deviation) for injection through 460-µm-thick rubber with different injection volumes and needles.

As evident from the high 10-second standard deviation in Table 1, we observed a varying degree of leakage at the injection site after extracting the needle after 3 s. For comparison, we therefore repeated the measurements with 460-µm-thick rubber patches, which presumably had higher structural stability than the scleral patches. Injection of isotonic saline through a rubber patch resulted in an initial, volume-dependent pressure pattern that was similar to that of a scleral patch. However, the pressure remained stable following extraction of the needle after 3 s, with almost no difference in pressure between the 3-second and 10-second timepoints. Compared with the standard 30-G needle, the four versions of the prototype needle decreased the peak pressure and increased the 3-second pressure drop. Table 2 and Fig. 5 show the pressure results for injection through 460-µm-thick rubber.

**Table 2:** Pressure results for injection through 460-m-thick rubber.

**50-µl injection volume**			
Needle type	Peak pressure, mean (SD) mmHg	Pressure after 3 s, mean (SD) mmHg	Pressure after 10 s, mean (SD) mmHg
Standard 30-G	57.3 (1.5)	53.7 (1.5)	52.7 (1.5)
One-groove prototype	50.3 (5.7)	40.3 (3.1)	40.0 (3.6)
Two-groove prototype	44.7 (3.2)	37.3 (3.8)	37.3 (3.8)
Three-groove prototype	37.7 (5.8)	30.7 (6.8)	30.7 (6.8)
Four-groove prototype	41.7 (5.1)	33.3 (5.5)	33.0 (5.2)
**70-µl injection volume**			
Needle type	Peak pressure, mean (SD) mmHg	Pressure after 3 s, mean (SD) mmHg	Pressure after 10 s, mean (SD) mmHg
Standard 30-G	67.3 (8.3)	63.3 (9.0)	62.7 (8.4)
One-groove prototype	51.3 (8.1)	41.7 (9.3)	41.3 (9.0)
Two-groove prototype	46.3 (5.9)	35.7 (5.5)	35.3 (4.9)
Three-groove prototype	43.8 (17.4)	35.0 (12.2)	34.5 (12.3)
Four-groove prototype	50.7 (1.5)	34.7 (1.2)	35.0 (1.0)
**100-µl injection volume**			
Needle type	Peak pressure, mean (SD) mmHg	Pressure after 3 s, mean (SD) mmHg	Pressure after 10 s, mean (SD) mmHg
Standard 30-G	89.3 (7.8)	80.7 (4.9)	79.7 (4.9)
One-groove prototype	64.3 (5.5)	46.7 (9.1)	46.7 (9.1)
Two-groove prototype	65.7 (8.0)	50.0 (5.0)	49.7 (5.5)
Three-groove prototype	58.3 (13.9)	50.0 (13.1)	48.7 (12.9)
Four-groove prototype	62.3 (9.1)	47.0 (8.5)	46.7 (9.0)

**Fig. 6:**
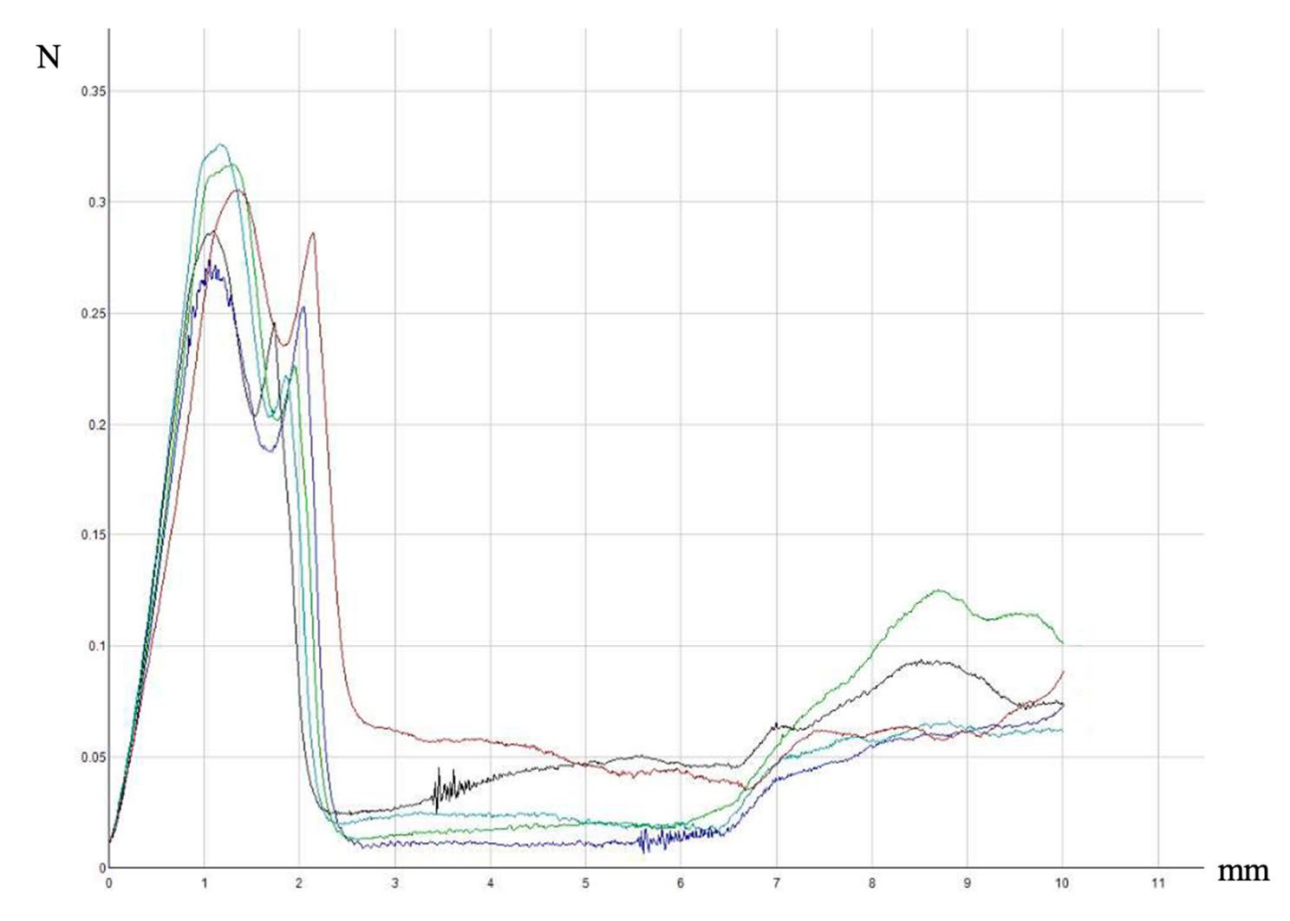
Load-displacement diagram of the four-groove prototype needle (five repetitions). The initial phases are similar to a standard needle. After 6-mm displacement of the needle, the force slightly increases, corresponding to the point at which the grooves reach the foil. This can be interpreted as a groove-mediated increase in friction along the needle shaft.

The DIN test showed that the initial phases of the prototype needle were similar to a standard needle. During the sliding phase, however, the force slightly increased after 6-mm displacement of the needle, corresponding to the point at which the grooves reached the foil. This can be interpreted as a groove-mediated increase in friction along the needle shaft, but we could barely feel this change in friction during the injections. Figure 6 shows the DIN test result for the four-groove prototype needle.

## Discussion

IVI transiently increases IOP, which might damage the optic nerve over time. In this in-vitro study we created an eye model to test a prototype needle for IVI that controls IOP by enhancing VR along grooves in the needle surface. Injection of isotonic saline through porcine sclera with a standard 30-G needle resulted in an initial, volume-dependent pressure peak. The pressure then dropped until we extracted the needle after 3 s and continued to fall gradually until the final measurement after 10 s. Injection with the prototype needle modified the pressure pattern by lowering the peak pressure and increasing the consecutive pressure drop, and each groove that was added to the prototype needle amplified this modified pressure pattern. Accordingly, the study provides proof of concept for a needle design that can mitigate the effect of IVI on IOP. The needle design can even be customized to match the injection volume of different biologics, e.g., a two-groove version for 50-µl injection volume and a four-groove version for 100-µl injection volume.

Because of the concern over an association between IVI-associated increase in IOP and RNFL loss, clinicians may choose to implement preventive measures to decrease IOP in connection with IVI. In this regard, there are two common prophylactic approaches: anterior chamber paracentesis (ACP) and IOP-lowering drugs (topical or oral acetazolamide). A systematic review and meta-analysis found an IOP-lowering effect of both approaches [22]. The study also found evidence for a protective effect of ACP on RNFL thickness. At the same time, both ACP and IOP-lowering drugs have drawbacks that may explain why neither is consistently used in IVI. ACP is an invasive procedure with risk of serious complications, such as hypotony, lens injury, and endophthalmitis. IOP-lowering medications, for their part, also have potential adverse reactions, they do not work immediately (which is unfavourable in a busy clinic), and there is lack of evidence that they actually prevent RNFL loss in IVI [22]. Accordingly, there is need for innovative solutions to control IOP in connection with IVI, and an IOP-lowering needle would have the benefit of being an integrated part of an otherwise standard IVI procedure.

The IOP-lowering effect of VR notwithstanding, the premise of many of the studies that we mentioned in the introduction is that VR is unwanted and, allegedly, leads to loss of the therapeutic agent. It has also been hypothesised that VR and incarceration could be a risk factor for post-injection endophthalmitis or induce rhegmatogenous retinal detachment [23, 24]. To the best of our knowledge, however, there is no strong evidence supporting these hypotheses. Several studies have indeed showed that only a very small amount of the injected drug is actually lost in connection with VR and that VR does not appear to diminish the therapeutic effect of IVI [7, 25–28]. Moreover, amid the frequent occurrence of VR, endophthalmitis and retinal detachment are in fact rare complications of IVI [29]. Taken together, VR in connection with IVI appears to be a common, harmless phenomenon that serendipitously lowers IOP. Still, we underscore that it was outside the scope of this study to assess possible side effects of the prototype needle, and that clinical research is warranted to study the safety and efficacy of enhancing VR to control IOP in IVI.

A recent study found a mean IOP immediately following intravitreal injections in patients of 50.7 mmHg for 50 µL-injection volume, 55.1 mmHg for 70-µL injection volume, and 62.3 mmHg for 100-µL injection volume [30]. In our study the equivalent peak pressure (standard needle) was 58.6 mmHg for 50-µL injection volume, 68.8 mmHg for 70-µL injection volume, and 86.0 mmHg for 100-µL injection volume. The somewhat higher modelled peak pressure in our study can have several explanations. First, our eye model did have the same volume as a human eye, but it was likely more rigid, which could have exaggerated the peak pressure. Second, the dynamic pressure transducer constantly measured the pressure, which allowed us to determine the peak pressure at the exact time of injection. By contrast, a few minutes probably pass before clinicians measure the post-injection IOP, allowing the pressure to decline slightly before measurement.

In addition to its in-vitro design, this study has some limitations that should be mentioned. First, we sometimes observed remarkable leakage at the injection site for porcine sclera after needle extraction. This observation was not only associated with the prototype needle but also the standard 30-G needle. While a standard 30-G needle often induces VR during IVI, we have not observed leakage that continues for several seconds after needle extraction in clinical practice. The leakage in this study was possibly due to desiccation or decomposition of some of the scleral patches, and it introduced a variation in the pressure measurements, which was most noticeable for the 10-second results. Moreover, while we used scleral patches in our eye model, conjunctiva may also contribute to limiting VR in vivo. Second, the scleral patches were not only from domestic pigs but also from animals that were much younger than most patients receiving IVI. Third, we did not design the experiment in a way that allowed us to differentiate drug reflux (represented by saline) and synthetic vitreous reflux, as both were clear fluids. Finally, the commercially available synthetic vitreous in this study was a homogenous fluid with a viscosity of 6–8 cP. This is about the same viscosity as porcine liquid vitreous, which, according to previous research, has a viscosity of 6.29 ± 2.3 cP [31]. However, the human vitreous liquefies with age, resulting in gel network collapse and separation into phases with different rheological properties [32]. Accordingly, we can expect more variation in VR and its effect on IOP in an in-vivo study due to the heterogenic, age-dependent composition of the human vitreous. Additionally, posterior vitreous detachment may influence VR [5].

In conclusion, this in-vitro study demonstrates proof of concept for a prototype needle that can mitigate the effect of IVI on IOP. Further research is necessary to study its clinical safety and efficacy.

## Data Availability

The authors confirm that the data supporting the findings of this study are available within the article.

## Abbreviations

anti-VEGF: Anti-vascular endothelial growth factor
IOP: Intraocular pressure
RNFL: Retinal nerve fibre layer
VR: Vitreous reflux
DIN: Deutsches Institut für Normung
SD: Standard deviation
ACP: Anterior chamber paracentesis
